# Psychological impact of being wrongfully accused of criminal offences: A systematic literature review

**DOI:** 10.1177/0025802420949069

**Published:** 2020-08-17

**Authors:** Samantha K Brooks, Neil Greenberg

**Affiliations:** Department of Psychological Medicine, King’s College London, UK

**Keywords:** Wrongful accusation, wrongful conviction, false allegations, exoneration, criminal justice

## Abstract

Being wrongfully accused of criminal offences can lead to serious negative consequences to those wrongfully accused and their families. However, there is little research on the psychological and psychosocial impacts of wrongful accusations. We conducted a systematic literature review to collate the existing literature, searching four electronic literature databases and reference lists of relevant articles. Data were extracted from 20 relevant papers, and thematic analysis was conducted on the data. Eight main themes were identified: loss of identity; stigma; psychological and physical health; relationships with others; attitudes towards the justice system; impact on finances and employment; traumatic experiences in custody; and adjustment difficulties. The psychological consequences of wrongful accusations appear to affect the lives of those accused seriously, even after exoneration or overturning of convictions. Strategies for improving public perception of wrongful convictions should be explored, and specific mental-health systems should be established to support those who are wrongfully accused.

## Introduction

There is a wealth of literature on the psychological impact on criminals post-conviction.^1^ However, there is far less research involving those who are wrongfully accused of a crime and later shown to be innocent, most probably because finding truly innocent individuals post-conviction is difficult. Furthermore, it is not unreasonable to assume there is an extra layer of resentment, frustration, confusion, anger and dissonance involved when the individual knows they were wrongfully accused.

Although there is disagreement about the frequency of wrongful accusations and convictions,^2^ a recent study estimated that wrongful convictions occur in 6% of criminal convictions leading to imprisonment.^3^ Other research estimates that up to 15.4% of all convictions are wrongful.^4^ There has been a recent increase in exonerations, related to technological developments (e.g. more sophisticated forms of DNA technology) and political developments.^5^ A wealth of literature has brought into question the accuracy of eyewitness identification,^6–8^ the potential suggestibility of children, adolescents and vulnerable adults, which can lead to false accusations and false confessions,^9,10^ and the use of coercive interrogation causing false confessions under duress.^11^ It has also been demonstrated that there is racial and class bias involved in wrongfully accusing people of crime and that these biases can influence decisions of guilt.^12,13^ A recent overview of wrongful convictions in Germany found that false allegations, eyewitness misidentification, false confessions and incorrect expert testimony contributed to wrongful conviction.^14^ In the UK, concern over miscarriages of justice led to the creation of an independent statutory body to investigate claims of miscarriages of justice – the Criminal Cases Review Commission (CCRC) was established in 1997.^15^ In the USA, a non-profit organisation called the Innocence Project is committed to exonerating wrongfully convicted individuals through DNA testing and, in 2019, reported that 362 people in the USA previously convicted of serious crimes had been exonerated since the founding of the project in 1992.^16^

Anecdotal reports of high-profile cases are commonplace in the media, such as the recent cases of footballer Ched Evans who was wrongfully accused of rape,^17^ care worker Gareth Jones who was imprisoned but later cleared of sexual assault^18^ and teenager Jay Cheshire who took his own life after being acquitted of rape.^19^ One high-profile case in the UK is that of Christopher Jefferies who was arrested for murder and released without charge, but not before being vilified by the press. Jefferies describes his experience as painful and his identity as ‘vandalised’, likening the experience to psychological torture.^20^ However, there are few peer-reviewed studies on the consequences of wrongful accusation. It is not unreasonable to assume that false accusations could have severe psychological consequences for those accused; false accusation has been compared to trauma experienced by military veterans, refugees, disaster survivors and prisoners of war.^21–23^ It has been suggested^24^ that there is a unique form of post-traumatic stress disorder (PTSD) experienced only by those wrongfully imprisoned. It is also reported that those wrongfully convicted and imprisoned can experience difficulties finding employment due to lacking job skills and the stigma surrounding their conviction,^25,26^ which can exacerbate mental-health problems.

This is the first systematic review of the literature on the psychological consequences of being wrongfully accused of a crime, as well as indirect consequences which may impact on psychological well-being (e.g. financial impact). In carrying out this review, we have not assumed that anyone who claims they were falsely accused is telling the truth. The research studies reviewed here involve participants who have been legally found not guilty or have had their convictions overturned. We also acknowledge it can be difficult for researchers to explore this important topic sensitively, and it remains highly important that true victims of crime should never be made to feel that their experiences have been demeaned.^27^

## Method

To be considered for inclusion in the review, studies were required to be published in English and to report on the psychological impact of wrongful allegations or an indirect impact which may have psychological consequences. Participants had to have been wrongfully accused (not necessarily convicted or charged) of a crime involving a victim (e.g. crimes such as abuse, sexual assault and murder; studies on crimes such as scientific misconduct were excluded, as the crimes are not comparable, and thus the impact of accusations may also not be comparable). Articles from online reports in addition to peer-reviewed journals were eligible for inclusion. However, grey literature, book chapters, dissertations, conference abstracts, commentaries and letters were excluded.

The search terms false allegation* OR false accusation* OR falsely accused OR false claim* OR wrongful conviction* OR wrongfully convicted OR wrongful accusation* OR wrongful allegation* OR exoneration OR exonerate* were used to search the following online databases: Embase, Medline, PsycInfo and Social Policy and Practice. Databases were searched from date of inception to April 2019, and then searched again in May 2020.

All citations found as a result of the search were exported to EndNote where duplicate papers were removed. The titles were then evaluated for their relevance to the topic of the review, and any which were clearly not relevant were excluded. Using the inclusion criteria, the abstracts were screened next, again removing any clearly not relevant to the review. The full-text articles of remaining citations were retrieved and read in their entirety, and again those not relevant for the review were excluded. Finally, reference lists of all remaining citations were hand searched.

A spreadsheet was designed to extract relevant data systematically from each paper. Data extracted from every paper included author names; year of publication; country of study; design; number and key characteristics of participants; measures used; and main findings related to the psychological impact of wrongful accusations. Thematic analysis^28^ was carried out on the papers’ results to identify recurring themes in the data and to develop a typology of results. Study results were coded by hand into themes by one author, and discussed between both authors.

## Results

A total of 2921 citations were found through the initial database search, and 54 duplicates were removed. Of those remaining, 2546 were excluded based on their title, 279 were excluded based on abstract and 24 were excluded based on full text. Two papers were unavailable online. An additional three papers were found via hand searching reference lists. This left 19 papers with findings relevant for this review. The searches were repeated in May 2020 to check for new publications. An additional 186 papers were screened, and one was included in the review, making a total of 20 papers. A summary of the characteristics of the papers can be seen in Table 1.

**Table 1.** Summary of papers.

Authors, year	Country	Measures	Participants
Alexander-Bloch et al., 2020	USA	PCL-5, PHQ-9, GAD-7, Pittsburgh Sleep Quality Index to assess post-traumatic stress disorder, depression, anxiety and sleep problems respectively	13 (100% male) exonerated ex-prisoners; 6 were exonerated for murder charges, 5 for sexual offences, 2 for armed robbery or ‘other’ offences
Burnett et al., 2017	UK	Interviews, written accounts and focus groups	30 (83.3% male) who had been falsely accused but with status of legal innocence
Campbell et al., 2004	Canada	Interviews	5 (100% male) who had been wrongfully convicted and imprisoned
Chinn and Ratliff		Interviews	11 wrongfully convicted and later released
Denov and Campbell, 2005	Canada	Interviews	5 (100% male) who had been wrongfully convicted and spent time in prison
DeShay, 2016	USA	Interviews (part of a wider study)	23 (88.9% male) who had been exonerated and released from prison
Grounds, 2004	UK	Descriptive study	18 (100% male) released from long-term imprisonment after convictions quashed on appeal
Hoyle et al., 2016	UK	Interviews, focus groups and written accounts	30 people who had been accused of abuse, all with the status of ‘legal innocence’ (i.e. not been charged, acquitted, or had conviction overturned); included 12 teachers, 8 approved school/community care workers, 3 in a religious role, 2 specialist home care workers and 5 ‘other’
Jarreta et al., 2004	Spain	Case report	1
Jenkins, 2013	UK	Longitudinal interview/observation study	132 (27 primary victims of wrongful conviction; 105 secondary victims)
Jenkins, 2014	UK	Longitudinal interview/observation study	132 (27 primary victims of wrongful conviction; 105 secondary victims)
Konvisser, 2015	USA	Interviews; pre-interview questionnaire developed for the study	21 (100% female) wrongfully convicted, and then exonerated
Page, 2013	USA	Focus groups as part of an evaluation of a financial training programme for exonerees	14 (100% male) exonerees
Pillai, 2002	UK	Qualitative	22 families who became subject to criminal or civil proceedings when a female adolescent/young adult developed a mental-health problem
Plumridge et al., 2016	UK	Qualitative interviews (phase 2 of a wider study)	30 foster carers who had experienced allegations of abuse/neglect
Rees, 2010	UK	Interviews	10 care workers from mental health/learning disability residential services who were suspended following an adult protection allegation but later exonerated
Schultz, 1989	USA	Cross-sectional study involving a study-specific questionnaire	100 (92% male) falsely charged with child sexual abuse
Westervelt and Cook, 2008	USA	Interviews	18 death row exonerees (4 experienced the trauma of confronting a death in the family while being wrongfully tried)
Wildeman et al., 2011	USA	Interviews; Before and After Exoneration Questionnaire; HADS and PCL-C	55 (100% male) exonerated after wrongful conviction and imprisonment
Zeman, 2005	USA	Literature searches of legal testimonies and online postings; qualitative interviews	63 parents whose children had been removed due to false accusations of abuse and neglect

Overall, the psychological impact of being wrongfully accused of a crime was described as extreme and long-lasting. Many negative impacts were reported, with various problems appearing to interact and compound each other. Eight key themes were identified, which are described below.

### Change in self-identity

Two papers reporting data from the same group of participants^27,29^ found that of the 30 participants, 19 felt they had experienced permanent changes to their personality, such as becoming paranoid and anxious (60%), hypervigilant or antagonistic (50%) and less confident (53.3%). Grounds^15^ found that 14/18 participants met the ICD-10^30^ criteria for ‘enduring personality change following catastrophic experience’, while families of the accused in the same study described their family member as being like a different person. Other changes included rejection of altruism and no longer wanting to help people^29^ and becoming hostile and mistrustful.^15^

Along with changes in personality, participants also experienced various other losses related to their sense of self, for example loss of dignity and credibility,^31^ loss of image of the self as a doting parent^32^ and loss of hope and purpose for the future.^15^ The consequence of the various personality changes and losses appeared to summate as a sense of loss of the pre-accusation self.^31^ It was particularly difficult for participants to regain their previous sense of self if they received no formal apology or public statement of innocence effectively ‘delabelling’ them.^33^

One paper^34^ noted positive changes in personality. Participants in this interview study noted that they had reflected on and grown from their experiences and reported changes such as a more positive attitude and not taking things for granted.

### Stigma

#### Damage to reputation

Two qualitative studies of the same group of participants^27,29^ reported that the vast majority (29/30 participants) reported damaged reputations or feeling stigmatised by others. Another^33^ found that participants struggled with managing stigma and suspicion from others, and Zeman^32^ reported that many participants felt their standing in the community had been affected negatively. Denov and Campbell’s^31^ and Chinn and Ratliff’s^21^ participants reported feeling labelled and vilified by others, while Rees^35^ found that 6/10 accused participants felt labelled as guilty by others both during and after the investigation. Many of Konvisser’s^34^ 21 participants reported that others assumed they were guilty; several reported that friends avoided them and strangers harassed them.

#### Self-stigma

As well as stigma from others, studies suggested that self-stigma (from within the individuals themselves) was also a concern. Burnett et al.^27^ concluded this was due to a combination of abhorrence at what they had been accused of and their inability to clear their name fully. In this study, 10/30 participants reported blaming themselves for the accusation, and struggled between wanting to fight the allegations and wanting to isolate themselves due to shame. Zeman^32^ also found that participants reported feelings of shame, blame and guilt. Similarly, Plumridge and Sebba^36^ reported that participants experienced feelings of guilt due to feeling ‘guilty until proven innocent’.

### Psychological and physical health

Data included in this theme revealed a perceived extreme impact on health, particularly mental health, often leaving participants unable to continue their normal work and social activities.^37^ Generally, such symptoms occurred in people without previous psychiatric histories, inferring such health problems are likely directly attributable to the wrongful arrest, conviction or imprisonment.^15^

#### Depression

Many studies reported that participants experienced symptoms of depression and suicidal ideation. Burnett et al.^27^ and Hoyle et al.^29^ found that 23/30 participants reported depression, with eight of these also reporting suicidal thoughts. In Grounds’ study,^15^ 10/18 participants suffered depressive disorders post imprisonment, and 14 suffered depressive episodes while in prison. Alexander-Bloch et al.^38^ found that 46% of their 13 participants reported moderate or high levels of depression. Campbell and Denov^39^ reported that all five of their participants had contemplated suicide at some point during their incarceration, with one making a suicide attempt. Wildeman et al.^40^ reported that 24/55 participants scored above the diagnostic cut-off score for depression. Symptoms of depression were also noted in several other studies.^34,35,37,41–44^

#### Anxiety

Anxiety and panic disorders also appeared to be common: 18/30 participants in the study reported by Burnett et al.^27^ and Hoyle et al.^29^ suffered anxiety or panic attacks. Grounds^15^ found that 27.8% of 18 participants reported features of panic disorder post incarceration, while 38.9% had features of panic disorder during their time in prison. Of the 55 participants in Wildeman et al.’s^40^ study, 22 met the criteria for anxiety, as did 46% of Alexander-Bloch et al.’s^38^ participants. Symptoms of anxiety and panic disorders were noted in four other studies.^34,35,41,42^

#### PTSD

Several papers also noted probable PTSD in those wrongfully accused: 17/30 participants in the study reported by Burnett et al.^27^ and Hoyle et al.^29^; 12/18 participants in the Grounds^15^ report; 42% of Alexander-Bloch et al.’s^38^ participants; and 15/55 participants in Wildeman et al.’s^40^ study. Konvisser^34^ also noted that many of the participants in their qualitative study reported experiencing PTSD.

#### Sleep problems

The study reported by Burnett et al.^27^ and Hoyle et al.^29^ found that 12/30 participants reported disrupted sleep, insomnia or nightmares. All participants in Schultz’s^43^ study reported some degree of sleeplessness or night terrors. Most participants reported on by Grounds^15^ had chronic difficulties sleeping. Jenkins^42^ found that many participants suffered from insomnia, Alexander-Bloch et al.^38^ found that 80% of 10 participants reported sleep problems occurring more than once a month, and Pillai^37^ reported many participants had developed sleep disorders.

#### Other psychological and somatic symptoms

Burnett et al.^27^ and Hoyle et al.^29^ reported a general sense among their participants of being ‘worn down’ by wrongful accusations and worried about others believing them. Almost half reported physical symptoms such as pain, high blood pressure and dietary problems. Other problems reported include unusual weight loss or gain,^43^ nausea,^43^ moodiness and irritability,^15,42^ somatic (physical) complaints,^35^ paranoia,^15,34^ stress^37^ and alcohol or drug dependency/misuse.^15,35^ Negative emotions included feelings of bitterness and unresolved feelings of loss,^15^ hopelessness,^15,45^ emptiness,^15^ anger and aggression,^31^ helplessness,^33^ chronic feelings of threat and fear when out in public^15^ and a sense of ‘survivor’s guilt’ if survivors of situations in which others had died.^33^

### Relationships with others

#### Isolation

In the study described by Burnett et al.^27^ and Hoyle et al.,^29^ 26/30 participants reported becoming socially withdrawn and isolated since their wrongful accusation, often due to a sense of alienation or deliberate withdrawal due to fear of being burdensome to others. DeShay^46^ also commented that participants reported withdrawing from other people and struggling to adjust to being around others. Grounds^15^ noted a difficulty adapting to life out of prison, reporting that participants used strategies of withdrawal, isolation and uncommunicativeness. Westervelt and Cook^33^ reported on participants’ apathy about maintaining close relationships.

#### Strain on relationships

Commonly, social networks, friendships and relationships appeared to break down after individuals were wrongfully accused. In the Burnett et al.^27^ and Hoyle et al.^29^ study, 27/30 participants reported a fractured social network, 17/30 reported a strain on intimate relationships and 8/30 reported a strain on relationships with children or grandchildren. Additionally, many reported feeling ‘forced out’ of friendships. Konvisser^34^ reported similar findings, with all 21 participants reporting a negative impact on their families, and changes in relationships with partners, children, parents or friends. Zeman^32^ also reported a strain on relationships with children, with those wrongfully accused experiencing a loss of the feeling they could protect their child and loss of the sense of a normal parenting experience. Rees^35^ and Schultz^43^ both reported that several participants had experienced family stress, relationship break-ups, divorce or loss of custody of children. Participants also reported the loss of family members who did not believe in their innocence,^31^ loss of friends,^42^ fractions and estrangement within families^41^ and relationship break-ups as well as conflicts with family which arose upon release from prison because families had adapted to living without them.^15^ Only one study^43^ reported a positive impact on relationships with others in a minority of cases: 8/100 participants in this study reported the family had been brought closer together after the acquittal as they fought an ‘outside enemy’.

#### Families experiencing strain and stigma

Several studies reported ‘secondary trauma’ in the close families of those wrongfully accused. Burnett et al.^27^ and Hoyle et al.^29^ reported cases of partners and families of the accused experiencing anxiety and depression, and Grounds^15^ also reported depression in the wives of those accused. Families also had to deal with the stigma and shame involved,^27,29,41^ which could lead to them being socially rejected, blamed, labelled and stereotyped by others in the community, and could even lead to antisocial or criminal behaviour in the children of the wrongfully accused.^41^ Grounds^15^ found that wives of the accused often felt unable to meet the needs of their children, and 9/30 participants in the study reported by Burnett et al.^27^ and Hoyle et al.^29^ felt their children’s lives had been disrupted.

### Attitudes towards the justice system

In the Burnett et al.^27^ and Hoyle et al.^29^ study, 28/30 participants reported loss of faith in the criminal justice system, while 20/30 reported loss of trust in the police. Similar disillusionment was noted by Campbell and Denov,^39^ whose participants reported intolerance of injustice, cynicism and mistrust in the fairness and legitimacy of authority figures following release from prison. Jenkins^41^ found that both primary and secondary victims of wrongful accusation experienced feelings of bitterness and resentment at the state not acknowledging the injustice or offering an apology. Konvisser’s^34^ participants reported a lack of apology or official acknowledgement of their innocence from prosecutors left them feeling as though they were under a cloud of guilt and could not obtain closure. Participants also reported fear and tension surrounding governmental entities,^21^ frustration at feeling betrayed by the system,^21^ loss of faith in the justice system^32^ and anger towards the system.^36^

Hoyle et al.’s^29^ participants reported both a loss of confidence in public opinion and fear of further allegations. Other fears included being retargeted by the criminal justice system,^31^ repeat accusations^33^ and further police involvement, imprisonment or children being removed.^36^

### Impact on finances and employment

#### Financial impact

In Burnett et al.’s^27^ and Hoyle et al.’s^29^ study, 28/30 participants reported a significant financial burden (despite having received legal aid and damages). Participants reported losses of up to £50,000 in legal fees and larger amounts for loss of earnings. Other financial issues reported in this study included reduced pensions, loss of homes and added financial pressure on partners. DeShay^46^ and Chinn and Ratliff^21^ reported a substantial financial impact on participants: all (*N*=23 and *N*=11, respectively) had to rely on family, friends or attorneys for financial support and housing. Jenkins^42^ found that 10/27 ‘primary victim’ participants were financially destitute. Pillai^37^ reported that the costs incurred to participants ranged from £10,000 to £100,000 in lost income and legal costs. In Schultz’s^43^ study, 12/100 felt bail was set too high for the average family income, and those who did not have property that could be posted as bail were concerned for their safety in jail or said that the money they should have used for defence costs was used up for bail. Further, 24/100 had applied for some sort of benefits, and 28/100 had to sell the family home to meet legal costs. Grounds^15^ reported that post imprisonment, participants had little sense of the value of money, which led to difficulty budgeting, reckless spending and debt. Additionally, Page^45^ found that participants reported problems such as overspending, not having enough money for basic needs, not having enough money to retire and frequent use of loans.

#### Employment

In the study reported by Burnett et al.^27^ and Hoyle et al.,^29^ most of the participants who were working at the time of their accusation (exact number not reported) had lost their jobs, been stripped of regular duties or faced impassable barriers against working with children or vulnerable adults in the future. Several reported struggling to get references from former employers, distress at no longer having a clean criminal record check and bitter feelings of loss about not being able to continue in their careers. DeShay^46^ reported that only 4/23 participants were employed, and all but one of those had started their own business, as they had experienced such difficulty finding other employment. In Pillai’s^37^ study, criminal prosecutions had resulted in suspension from work for 5/22 participants, with two unable to return to work at all. Of 100 participants in Schultz’s^43^ study, 82 had suffered some type of job loss or penalty. Several of Konvisser’s^34^ participants reported that they were unable to find a job, often because of the stigma associated with their conviction, leading to financial struggles. There were additional problems when the false accusation was related to the participant’s employment. For example, Burnett et al.^27^ found that 23/30 participants reported feeling anger at their employers. Rees^35^ interviewed 10 practitioners who had been accused of wrongdoing at work and who were subsequently exonerated. Participants reported a long, difficult and confusing process in which they were not made aware of the nature of the allegations until late in the process, were refused access to the workplace and were forbidden from contacting colleagues. All felt unsupported and isolated during the process, with many reporting anger and bitterness towards their employers. Lack of information and delays caused great distress to the accused and their families, with 8/10 stating that waiting and stress caused self-doubt and rumination about the reasons for their suspension. Participants perceived that there were no routes for redress following exoneration, and six reported ongoing anxiety after returning to work.

### Traumatic experiences in custody

Several studies involving people who had been wrongfully imprisoned found that traumatic experiences during the imprisonment appeared to contribute to post-release problems. For example, Grounds^15^ found that 14/18 participants experienced fear of being assaulted or killed in prison; three had been subjected to serious violence. Several reported having learned to be aggressive and intimidating as a form of self-protection. Participants also reported assaults, threats and sleep deprivation in police custody. Chinn and Ratliff’s^21^ participants all reported experiencing some level of police misconduct, with several describing it as a dehumanising experience. Jenkins^42^ reported that participants found their cross-examination hostile and combative to the point of leaving them feeling traumatised. Many of them continued to have nightmares about their police questioning, and reported feeling intimidated, bullied and, in 2/27 cases, physically abused by police officers. Their experiences during the criminal trial continued to affect them, with several reporting panic attacks relating to their court experiences. Violence in prison, or fear of violence from other inmates, was also reported in three other studies.^21,42,45^

Konvisser’s^34^ participants reported intimidation and harassment from the police and in prison; several felt traumatised by the way police had treated them. They described the prison experience as degrading, demeaning and traumatic, and reported feeling lonely and powerless. Several participants had been treated as suicide risks by prison staff, despite not feeling suicidal, which was humiliating and confusing.

Campbell and Denov’s^39^ participants reported a violent and hostile environment in prison, including harassment and intimidation by other inmates. This study identified problems during imprisonment which may be unique to those wrongfully accused: preoccupation with proving innocence tended to be seen by the prison administration as evidence of a lack of remorse and inability to adapt to the prison environment; maintaining one’s innocence in prison seemed to lead to the administration seeing them as a higher risk for reoffending, leading to them being ineligible to cascade to lower security institutions and curtailment of privileges such as seeing family and prison activities.

### Adjustment difficulties

In many cases, participants’ legal battles had been ongoing for years, and the experience of being wrongfully accused (and in some cases, convicted and imprisoned) had dominated their lives for long periods of time. Those who had been incarcerated tended to become institutionalised and struggled to adapt to life out of prison.^42,45^ Denov and Campbell^31^ reported participants experienced panic and self-consciousness around everyday tasks following their release from prison, while Konvisser^34^ also found that participants reported difficulties adjusting to having freedom again and often took on too much to cope with in their efforts to move on. Grounds^15^ found that participants reported difficulty coping with ordinary tasks in the initial weeks after their release but felt humiliated by this and ashamed to ask for help. Many felt unsettled, could not find a sense of direction, struggled to reintegrate back into their families and experienced difficulties coping with cultural changes that had taken place since their conviction. Grounds^15^ refers to these adjustment problems as being ‘dislocated in time’ (p. 172), suggesting that participants were ‘developmentally frozen’ as they still felt the age they had been when they went to prison, whereas the world and their families had moved on without them. Similar findings were reported by Chinn and Ratliff,^21^ whose participants described feeling frozen in time while their families and friends had moved on with their lives. They explained this as a ‘culture shock’ on release, whereby they did not understand cultural changes that had taken place during their incarceration and felt behind in technology and knowledge.

## Discussion

The impact of wrongful accusation is frequently complex and long-lasting, with participants reporting negative impacts on their self-identity, reputation, psychological and physical health, relationships with others, attitudes towards the justice system, finances and adjusting to life after their convictions were overturned. These problems appeared to compound each other and exacerbate the psychological difficulties experienced by the wrongfully accused. Negative impacts on family members were also reported, with those close to the wrongfully accused also experiencing stigma and psychological difficulties. For those who were wrongfully imprisoned, traumatic experiences during their incarceration exacerbated the psychological difficulties they faced.

It is important to note that the nature of wrongful accusations differed between studies. We included studies of any participants who had experienced a wrongful allegation. In some cases, these were accusations at work, leading to suspension and investigation, while others were investigated through the criminal justice system and in many cases imprisoned. However, similar themes emerged from all papers.

This review shows that the consequences of wrongful accusations can be severe. Consequently, it is crucial to identify the best ways to support those who are wrongfully accused and their families. There is some evidence that activism can be helpful: many exonerees feel compelled to seek out ways to understand their experiences and help others going through the same thing such as becoming involved in the policy reform process and engaging in education, raising awareness and advocacy. Such activities might help them find meaning in their own experience, normalise the trauma and rebuild their confidence and sense of purpose.^15,34,47^ However, while this may be helpful for some people, it should always be their own choice, and they should never be ‘forced’ into activism or speaking engagements, as while some find this healing, others may just want to ‘move on’^47^ or could find that it triggers negative emotions.^34,48^

It is likely that having a support network of people who understand the unique experience of being wrongfully accused and who do not judge can help the wrongfully accused feel able to recover from their experience. For example, Jenkins^42^ found that attendance at miscarriage of justice support groups provided support and empathy, and Konvisser^34^ found that participants highly valued meeting others in the same position at conferences and events aimed at discussing miscarriages of justice. We suggest that further work is required to help to understand better what constitutes an effective support networks in terms of allowing wrongfully accused/exonerated people to find and support each other. It is also possible that such groups could have a negative impact on some individuals. So, more research is needed to explore this. Studies have also shown that support from close others can also be protective: Campbell and Denov^39^ found that the support of family and loved ones was cited as a reason for not attempting suicide, and Konvisser^34^ also noted that support from close others was protective of psychological well-being. However, this review found that relationships with loved ones are often fractured during the process, and so the support available to the wrongfully accused was damaged. It is particularly important, then, that those without family support have access to support in other ways, for example miscarriage of justice support groups or simply new social groups.

There is some evidence of post-traumatic growth occurring after wrongful convictions are overturned in a minority of those affected.^22^ This supports the findings of research on other traumatised populations such as those affected by disasters,^49^ who report positive consequences of trauma such as greater appreciation of life and relationships. Post-traumatic growth tends to be associated with both social support and effective coping strategies,^49^ and it would therefore be useful to explore the best coping strategies for the wrongfully accused to use. For example, while support groups and activism may be useful strategies, these are not helpful for everyone, and so there may be other coping mechanisms (such as forming new social networks, exercise, mindfulness or yoga) which work better for people who do not wish to become involved in these. Future research should explore different ways of coping. Aslan^50^ suggests that those falsely accused of sex offences tend to adopt more emotion-focused than task-focused coping strategies, which is less constructive as it deals with the emotional affect rather than trying to problem solve. Future research could explore why this might be and take into consideration the effect of such coping strategies on psychological well-being.

Also of importance is improving public perceptions of those wrongfully accused. Research suggests public perceptions of exonerees tend to be negative and not dissimilar to perceptions of actual offenders, despite knowing they had been exonerated.^51–53^ This may be because the public are concerned that the exoneration process itself was flawed, and they prefer to believe the veracity of the original conviction, despite the outcome of the legal review process.^54^ Future research should consider ways of improving public perception. Otherwise, the wrongfully accused will continue to be stigmatised by others, which is likely to worsen the psychological impact of their experience. Weigand^48^ suggests that more public education (e.g. about the causes and impact of wrongful conviction) for communities could provide exonerees the support they need, and that limiting ‘sensationalism’ in stories of wrongful conviction may help improve public perception.

There may also be a need for policy reforms in order to minimise both the potential for wrongful conviction and the impact experienced by those who are wrongfully accused. Weigand,^48^ based on her own experience in advocacy for exonerees, recommends increasing interaction and cooperation between health professionals and legal experts to ensure the exoneration process is as smooth as possible, as well as providing more funding for case management and mentoring services. Provision of bespoke, fast-track mental-health services, including vocational rehabilitation, would also be helpful. The literature also reveals important implications for the way prisoners are treated while incarcerated. For example, Campbell and Denov^39^ noted problems during incarceration unique to the wrongfully accused, with their preoccupation with proving their innocence actually leading the prison administration to see them as unremorseful and to limit their privileges. It may be the case that the prison administration should not use ‘remorse’ as a helpful indicator of anything, as this construct is problematic,^55^ and research is needed to explore whether remorse is actually associated with improved outcomes post release. Prison staff should be trained to deal with current risks in relation to privileges, rather than on the potentially unhelpful construct of remorse.

### Limitations

The data screening, extraction and analysis for this review were carried out by one researcher. Although both authors discussed the findings at each stage, it may have been useful for a second researcher to have repeated these stages in order to ensure that no relevant data were excluded. The literature search was limited to four databases, and although hand searches of reference lists were carried out to reduce the risk of missing relevant papers, it is possible that some were still missed, and a search of more databases may have yielded more papers.

In terms of the literature itself, almost all studies had small sample sizes. This is likely due to the very nature of the subject matter – the wrongfully accused are a hard-to-reach population, and it is difficult for researchers to locate cases, to be sure that those included in the study were indeed wrongfully accused and to access case materials.^5^ The highly sensitive nature of the research means that many wrongfully accused individuals may be unwilling to take part, and so the literature tends to be limited to those self-selecting to participate. There can be no random samples or control groups with research on this type of population,^5^ and results may not be generalisable to the larger wrongfully accused population.

## Conclusion

Research on the wrongfully accused is challenging, with academics facing not only a difficult-to-reach population but also the challenge of dealing with data of such a sensitive nature, needing to accept that justice is not always 100% right and highlighting the struggles faced by the wrongfully accused without allowing a guilty person’s distress to distract from justice or cause distress to the original victims of crime. However, we suggest that it is important to keep an open mind about innocence and guilt until the facts are clear, and to acknowledge that miscarriages of justice do happen for a multitude of reasons. The psychological impact on those wrongfully accused appears to be vast, severe and long-lasting. Our results suggest that appropriate systems need to be in place to support these individuals and their families. It is positive that there are avenues allowing for re-examination of cases (e.g. the CCRC), but overturning of original verdicts may need an allocation of specialist support services to help people readjust and to treat any mental-health disorders that have developed because of the injustice. Policy reforms, strategies for improving public perception of wrongful convictions, a change in the media’s portrayal of such cases and funding for specialist mental-health services and support groups specifically for wrongfully accused people may be helpful.
